# Aerospace stressors and brain health in military aviators: a glymphatic–neurovascular framework

**DOI:** 10.3389/fphys.2026.1953796

**Published:** 2026-09-21

**Authors:** Joel Ramirez, Vincent N. Marraffino, Ursula A. Anirud, Shawn G. Rhind

**Affiliations:** 1 Dr. Sandra Black Centre for Brain Resilience and Recovery, Sunnybrook Research Institute, Toronto, ON, Canada; 2 Department of Medicine, University of Toronto, Toronto, ON, Canada; 3 Graduate Department of Psychological Clinical Science, University of Toronto Scarborough, Toronto, ON, Canada; 4 Defence Research and Development Canada, Toronto Research Centre, Toronto, ON, Canada; 5 Faculty of Kinesiology and Physical Education, University of Toronto, Toronto, ON, Canada

**Keywords:** brain health, glymphatic system, hypobaria, military aviators, neuroimaging, neurovascular unit, sleep and circadian disruption

## Abstract

Military aviation exposes aircrew to interacting physiological and operational stressors that are often studied in isolation. These stressors are experienced by the whole body: pulmonary, cardiovascular, musculoskeletal, endocrine, and immune responses may influence cerebral perfusion, blood–brain barrier function, fatigue, and recovery. Evidence from aviation cohorts, altitude-chamber studies, and adjacent fields suggests that some exposures may converge on neurovascular, blood–brain barrier, perivascular, and inflammatory pathways before overt neurological disease is apparent. This narrative review evaluates the glymphatic system as one potentially important component of that broader injury-and-recovery network, rather than as a sole or established primary mechanism. We synthesize evidence concerning hypobaria and decompression, intermittent hypoxia and hyperoxia, high +Gz, sleep and circadian disruption, operational wakefulness-promoting agents, noise, cosmic ionizing radiation, and context-dependent chemical exposures. Direct human evidence of glymphatic dysfunction in tactical aviators remains absent, and findings from lower-intensity hypobaric exposure and retrospective clinical cohorts are mixed or null. Exposure profiles also differ substantially among fighter, cargo/tanker, helicopter, and remotely piloted aircraft communities. We therefore propose an exposure-resolved research agenda combining longitudinal neuroimaging, circulating biomarkers, electroencephalography, cognitive testing, sleep phenotyping, dosimetry, and controlled single- and combined-stressor experiments. This calibrated glymphatic–neurovascular framework generates testable hypotheses about cumulative brain stress and recovery while preserving alternative explanations involving direct tissue injury, cerebral perfusion, endothelial dysfunction, oxidative stress, systemic and central inflammation, and autonomic regulation.

## 1 Introduction

Military fighter aviation represents one of the most demanding occupational environments for the human brain. Tactical aviation platforms such as the CF-18 and F-35 repeatedly expose aircrew to a complex ensemble of physiological and operational stressors: high and dynamic G-forces, rapid altitude transitions, hypobaria, intermittent hypoxia or hyperoxia, autonomic strain, heavy cognitive workload, sleep restriction, and circadian disruption linked to global mobility and transmeridian operations (Summerfield et al., 2018; Tornero-Aguilera et al., 2025). Although the acute effects of individual stressors have been studied, their cumulative and interactive long-term impacts on neurovascular integrity and brain waste clearance remain poorly understood, and are rarely considered together from a systems physiology perspective (Estrella et al., 2026).

The extreme conditions of tactical and space aviation are not brain-limited exposures. Hyperoxia and dry breathing gas challenge the respiratory system; +Gz and anti-G straining impose cardiovascular and musculoskeletal loads; hypobaria affects gas exchange and vascular function; and sleep loss, heat, vibration, workload, and psychological stress engage endocrine and immune pathways. The brain is therefore situated within a systemic exposure response, and peripheral vascular or inflammatory signals may modify BBB integrity, cerebral perfusion, neurovascular coupling, and recovery. A brain-health framework that ignores these extracranial responses risks misattributing a multisystem physiological burden to a single intracranial mechanism.

Concern regarding the neurological impact of aviation stress is not new. Historically, long-term neurological research in tactical aviation has focused predominantly on spinal disorders, leaving the long-term cognitive and neurovascular consequences of repeated high-G and hypobaric exposure comparatively understudied (Drew, 2000; Temme et al., 2023). Military working groups have previously emphasized the need for prospective longitudinal studies in this population, but the substantial logistical and financial challenges of such research have led to reliance on cross-sectional designs and retrospective cohort analyses (Fear, 2021; Rice et al., 2026).

Despite these limitations, studies in military and high-altitude aviators have reported associations between occupational exposure and white matter hyperintensities (WMH), structural brain changes, and reductions in cognitive function (McGuire et al., 2014, 2016, 2019; Ramirez et al., 2024). WMH are areas of increased signal on T2/FLAIR brain MRI reflecting cerebral small vessel disease and white matter pathology; they are now recognized as common even in midlife and associated with measurable cognitive decline in the general population (Kloppenborg et al., 2014; d’Arbeloff et al., 2019). High-altitude exposure has also been linked histologically to tau protein deposition, neuroinflammation, and myelin loss, suggesting that repeated altitude stress may extend beyond reversible functional changes to chronic neuropathological sequelae (Iacono et al., 2022). Consistent with these observations, complementary neuroimaging and blood-based biomarker studies in a Royal Canadian Air Force (RCAF) cohort have identified converging evidence of brain alterations associated with repeated high-performance flight (Rhind et al., 2024; Ramirez et al., 2024, 2025; Mohamed et al., 2026).

One potentially unifying framework for these neurobiological observations is the *glymphatic system*, a brain-wide perivascular clearance pathway that facilitates exchange between cerebrospinal fluid (CSF) and interstitial fluid (ISF) to remove metabolic waste and injury-associated molecules from the central nervous system (CNS) (Iliff et al., 2012; Jessen et al., 2015; Plog and Nedergaard, 2018). Glymphatic function is strongly influenced by sleep, vascular pulsatility, astrocytic aquaporin-4 (AQP4), and meningeal lymphatic drainage; and, growing evidence implicates its dysfunction in traumatic brain injury (TBI), cerebral small vessel disease, aging, and neurodegenerative disorders (Louveau et al., 2017; Hablitz and Nedergaard, 2021; Jia et al., 2026). Although aspects of the glymphatic model remain under active investigation, it provides a useful systems-level framework for understanding how aerospace stressors may impair brain homeostasis.

Throughout this review, glymphatic impairment is treated as a candidate contributor within a broader neurovascular injury-and-recovery network—not as an exclusive or necessarily primary cause of aviation-associated brain changes. Direct tissue injury, BBB and endothelial dysfunction, altered cerebral perfusion, oxidative and inflammatory signaling, autonomic dysregulation, and impaired recovery may operate independently, upstream, downstream, or in parallel. The framework is therefore intended to organize testable relationships rather than to establish causal primacy.

Perivascular spaces (PVS) are fluid-filled channels surrounding penetrating cerebral blood vessels that support glymphatic transport and are recognized as neuroimaging markers of cerebral small vessel disease and putative indicators of glymphatic dysfunction when enlarged or visible on brain MRI (Ramirez et al., 2016; Wardlaw et al., 2020). This framework is particularly relevant to military fighter aviation, where hypobaria, intermittent hypoxia, repeated high gravitational (+Gz) exposure, sleep disruption, circadian misalignment, and operational stress are all biologically plausible disruptors of neurovascular and glymphatic homeostasis. Collectively, these stressors may impair brain clearance pathways, prolong neuroinflammatory responses, and contribute to both acute performance variability and cumulative neurological risk.

Support for a broader neurovascular-fluid dynamics framework also comes from spaceflight research. Human spaceflight and head-down tilt analogs have demonstrated alterations in CSF dynamics, cerebral perfusion, and circulating biomarkers consistent with disrupted brain homeostasis and metabolite clearance, despite the absence of overt neurological impairment (Schmidt et al., 2025; Estrella et al., 2026). Although microgravity analogs are not directly equivalent to fighter aircraft flight, these findings reinforce the concept that repeated perturbations of intracranial fluid regulation can produce measurable CNS biological signatures. An ocular glymphatic system has also been proposed to contribute to optic disc edema and spaceflight-associated neuro-ocular syndrome, further linking impaired CSF and perivascular fluid dynamics to neurological adaptations in extreme environments (Wostyn and De Deyn, 2018; Wostyn et al., 2020; Venegas, 2024).

To our knowledge, no published study has directly compared glymphatic surrogate markers between tactical aviators and appropriately matched non-tactical controls across an operational career. This knowledge gap is becoming increasingly important as fifth- and sixth-generation air combat introduces greater mission complexity, sustained cognitive demands, irregular operational schedules, and prolonged physiological stress. These evolving operational demands underscore the need to better understand the biological mechanisms that may contribute to long-term brain health in military aviators (Scott et al., 2013; Wand et al., 2023; D’Alessandro et al., 2024).

This review has three main objectives. First, we provide an overview of the glymphatic system, its relationship with sleep, and its interactions with blood-brain barrier (BBB) integrity and neurovascular function. Second, we synthesize current evidence suggesting that fighter-relevant aerospace stressors may converge to disrupt these interconnected systems through shared physiological mechanisms. Finally, we propose a translational research agenda incorporating multimodal neuroimaging and blood-based biomarkers to support longitudinal monitoring of brain health, recovery, operational performance, and the early detection of neurodegenerative change in military aviators.

### 1.1 Narrative review approach and scope

Because the relevant evidence spans aviation medicine, sleep science, neurovascular biology, environmental health, traumatic brain injury, and spaceflight, we used a targeted narrative-review approach rather than a formal systematic review. Iterative searches of PubMed/MEDLINE and general academic search engines, supplemented by backward and forward citation review of key papers and the authors’ source bibliography, were updated through 17 August 2026. Search concepts combined military aviation, aircrew, hypobaria, decompression, hypoxia, hyperoxia, acceleration, electroencephalography or EEG, cerebral perfusion, sleep, circadian disruption, aviation fatigue, modafinil, noise, cosmic radiation, PFAS or chemical exposure, systemic inflammation or cytokines, glymphatic or meningeal lymphatic function, BBB, PVS, WMH, DTI-ALPS, and neurological biomarkers. We prioritized human operational and chamber studies, then mechanistic animal work and adjacent-field studies when direct aviation evidence was unavailable. Null and equivocal findings were retained when they constrained interpretation. No PRISMA flow diagram, formal risk-of-bias instrument, or meta-analysis was undertaken; study selection was purposive and intended to support a critical, hypothesis-generating synthesis. During manuscript preparation and revision, ChatGPT and Codex (GPT-5; OpenAI, San Francisco, CA, United States) were used for language refinement, structural organization, citation cross-checking, and figure clarity; the authors verified all outputs and retained full responsibility for the manuscript.

## 2 Background

### 2.1 The glymphatic system: an overview

The brain lacks a conventional intraparenchymal lymphatic network yet must continuously remove metabolic by-products, excess ISF, and potentially toxic proteins. The glymphatic system is a glial-associated perivascular clearance pathway in which CSF enters the brain along periarterial spaces, exchanges with ISF, and exits through perivenous pathways and meningeal lymphatic vessels (Iliff et al., 2012; Jessen et al., 2015; Plog and Nedergaard, 2018; Jia et al., 2026). This exchange is facilitated by astrocytic endfeet enriched with AQP4 water channels, which regulate perivascular fluid movement and waste clearance (Iliff et al., 2012; Jia et al., 2026). Effective glymphatic transport depends not only on AQP4 expression but also on its polarized localization within astrocytic endfeet adjacent to the cerebral microvasculature. Meyerhoff et al. (2022) emphasized the particular relevance of this organization to military neuroscience, noting that injury-associated loss of perivascular AQP4 polarization may impair glymphatic transport, promote cerebral edema, and compromise neurological function. Impaired glymphatic function has been linked to reduced clearance of neurotoxic proteins, neurovascular dysfunction, cognitive decline, and neurodegenerative disease, while MRI-visible PVS have emerged as practical imaging markers of glymphatic dysfunction (Rasmussen et al., 2018; Nedergaard and Goldman, 2020). Recent syntheses place glymphatic redistribution within an integrated clearance network that also includes blood–brain barrier efflux, intramural periarterial drainage, and meningeal lymphatic outflow (Voumvourakis et al., 2026; Zahran et al., 2026). Although tracer experiments and human imaging observations support organized perivascular fluid transport, the magnitude, direction, and driving forces of transport through brain parenchyma remain debated, including the relative roles of advection, diffusion, vascular pulsatility, and sleep state (Abbott et al., 2018; Smith and Verkman, 2018; Wang et al., 2026). Accordingly, MRI-visible PVS and DTI-ALPS should be interpreted as indirect, non-exclusive surrogates rather than direct measurements of whole-brain clearance.

Sleep is a major physiological regulator of glymphatic function. During sleep, particularly non-rapid eye movement (NREM) sleep, CSF-ISF exchange and metabolic waste clearance are enhanced, whereas wakefulness, sleep deprivation, and elevated noradrenergic tone tend to suppress these processes (Mendelsohn and Larrick, 2013; Xie et al., 2013). Recent evidence suggests that locus coeruleus-driven norepinephrine oscillations during NREM sleep coordinate glymphatic CSF influx, highlighting the importance of normal sleep architecture rather than sleep duration alone (Hauglund et al., 2025). In a randomized crossover study of 39 healthy older adults, morning plasma Aβ and tau species were higher after normal sleep than after sleep deprivation; device-derived physiology and compartmental modeling were consistent with enhanced sleep-active brain-to-plasma clearance, although glymphatic transport was inferred rather than measured with a validated direct tracer (Dagum et al., 2026). Consequently, chronic sleep restriction and circadian disruption may impair brain waste clearance, while pharmacologically induced sleep may not fully replicate the restorative effects of natural sleep (Hauglund et al., 2025). These findings are particularly relevant to military fighter aviators, whose operational demands frequently compromise sleep opportunity, circadian alignment, and recovery.

Glymphatic clearance ultimately drains through the meningeal lymphatic system, which transports CSF-derived solutes, immune cells, and metabolic waste to the cervical lymphatic circulation (Louveau et al., 2017; Jia et al., 2026). Together, these pathways form an integrated brain clearance network that facilitates waste removal and the transport of CNS-derived proteins into the peripheral circulation (Plog et al., 2015; Da Mesquita et al., 2018). Consequently, circulating biomarkers such as amyloid-beta (Aβ), tau, glial fibrillary acidic protein (GFAP), and neurofilament light chain (NfL) may reflect not only CNS injury but also the efficiency of glymphatic-lymphatic clearance, an important consideration when interpreting blood biomarkers in translational and operational research (Plog et al., 2015; Dagum et al., 2026).

The glymphatic system operates within the broader neurovascular unit (NVU), where endothelial cells, pericytes, astrocytic endfeet, and the BBB coordinate CSF-ISF exchange and maintain cerebral homeostasis (Estrella et al., 2026). Efficient glymphatic clearance depends on intact BBB integrity and healthy neurovascular function, whereas BBB disruption impairs perivascular exchange, promotes neuroinflammation, and contributes to the accumulation of pathological proteins (Harrison et al., 2020; Ishida et al., 2022; Lopes et al., 2022, 2025; Estrella et al., 2026). Human imaging studies further support this relationship by linking BBB dysfunction with reduced glymphatic efficiency (Verheggen et al., 2018; Bhattarai et al., 2025; Hu et al., 2025).

Aerospace stress may reach this NVU from outside the CNS. Circulating cytokines can communicate with the brain through endothelial signaling, neural and humoral routes, and selective transport across the BBB; foundational work established that several cytokines traverse the BBB through regulated pathways rather than being excluded categorically (Banks et al., 1995). Inflammation can in turn alter endothelial junctions, astrocytic function, and perivascular homeostasis, while impaired clearance may prolong inflammatory signaling, creating a potentially bidirectional cycle (Cai et al., 2024; Zou et al., 2024). This mechanism is directly relevant to the whole-body aviation environment but remains a bridge hypothesis: systemic cytokine elevation in an aviator does not by itself demonstrate BBB leakage, neuroinflammation, or impaired glymphatic transport.

## 3 Aerospace stressors

### 3.1 Hypobaria and decompression

Military fighter aviators are routinely exposed to rapid changes in ambient pressure during high-altitude operations. Despite modern aircraft pressurization systems and life-support equipment, repeated exposure to hypobaria and decompression stress remains an inherent feature of tactical aviation (Sherman and Sladky, 2018; Connolly et al., 2024). These exposures can promote intravascular microbubble formation, endothelial activation, inflammatory signaling, and microvascular injury, with the potential to compromise BBB integrity even in the absence of overt decompression sickness (Hills and James, 1991; Yang et al., 2013). Increasing evidence from military aviation and altitude chamber studies suggests that these physiological perturbations may accumulate over repeated operational exposures, providing a biologically plausible pathway linking hypobaria to neurovascular dysfunction. **Table 1** summarizes the exposure-specific evidence and candidate pathways, and **Figure 1** depicts the proposed converging glymphatic–neurovascular network.

**Table 1:** Proposed neurovascular and glymphatic relevance of fighter-associated and context-dependent aerospace stressors.

Stressor	Primary physiological effects	Putative glymphatic and neurovascular impact	Potential neurological implications	Reference
Hypobaria/decompression stress	Intravascular microbubble formation, endothelial activation, inflammatory signaling, microvascular injury	Endothelial and neurovascular dysfunction, BBB disruption, impaired perivascular exchange and glymphatic clearance	WMH burden, neuroinflammation, cumulative neurovascular injury	Connolly et al., 2018, 2023b, 2024; McGuire et al., 2013
Intermittent hypoxia/hyperoxia	Oxygen-dependent cerebral vasoconstriction and perfusion reduction, oxidative and inflammatory signaling; responses vary by FIO2, delivery pattern, and pressure	Reduced cerebral perfusion reserve, endothelial and neurovascular stress, possible BBB and perivascular effects	Cognitive impairment, neurovascular injury, oxidative injury	Lambertsen et al., 1953; Damato et al., 2020, 2022a, 2024; Estrella et al., 2026
High +Gz	Venous pooling and transient cerebral hypoperfusion; time-resolved EEG changes during G-LOC and recovery	Neurovascular stress and impaired cerebrovascular regulation; no direct evidence of chronic glymphatic injury	Neuronal stress, cognitive impairment, neurovascular injury	Wilson et al., 2005; Bailey et al., 2020; Blacker and Dooley, 2024; Rhind et al., 2024
Sleep restriction/circadian disruption	Sleep loss, extended wakefulness, circadian disruption, workload, and potentially systemic inflammatory fatigue	Reduced glymphatic clearance, neurovascular dysfunction, BBB disruption, altered CSF dynamics	Slower recovery, metabolite accumulation, cognitive and performance impairments	Caldwell et al., 2009; Mendelsohn and Larrick, 2013; Damato et al., 2022b; Plog et al., 2015
High-intensity noise	Auditory injury, autonomic and stress responses, possible vascular and inflammatory effects	Lower DTI-ALPS and higher free water reported in non-aviation NIHL; causality unestablished	Hearing loss, fatigue, cognitive load; candidate perivascular association	Wang et al., 2026
Cosmic ionizing radiation	Cumulative low-dose exposure varying with altitude, latitude, duration, and solar activity	Direct aviation glymphatic evidence absent; BBB effects suggested only in indirect/combined animal models	Context-dependent modifier; neurological relevance at aviation doses uncertain	Bellone et al., 2016; Scheibler et al., 2022
PFAS and other chemical exposures	Exposure varies by firefighting foam, treated materials, fuels, maintenance tasks, base, and era	Experimental PFOS-related endothelial–astrocyte and BBB disruption; no direct aviation glymphatic data	Plausible vascular co-exposure; aviation-specific brain risk unestablished	Yu et al., 2020

Evidence strength varies substantially; rows summarize hypotheses and indirect findings, not established causal effects.

**Figure 1:**
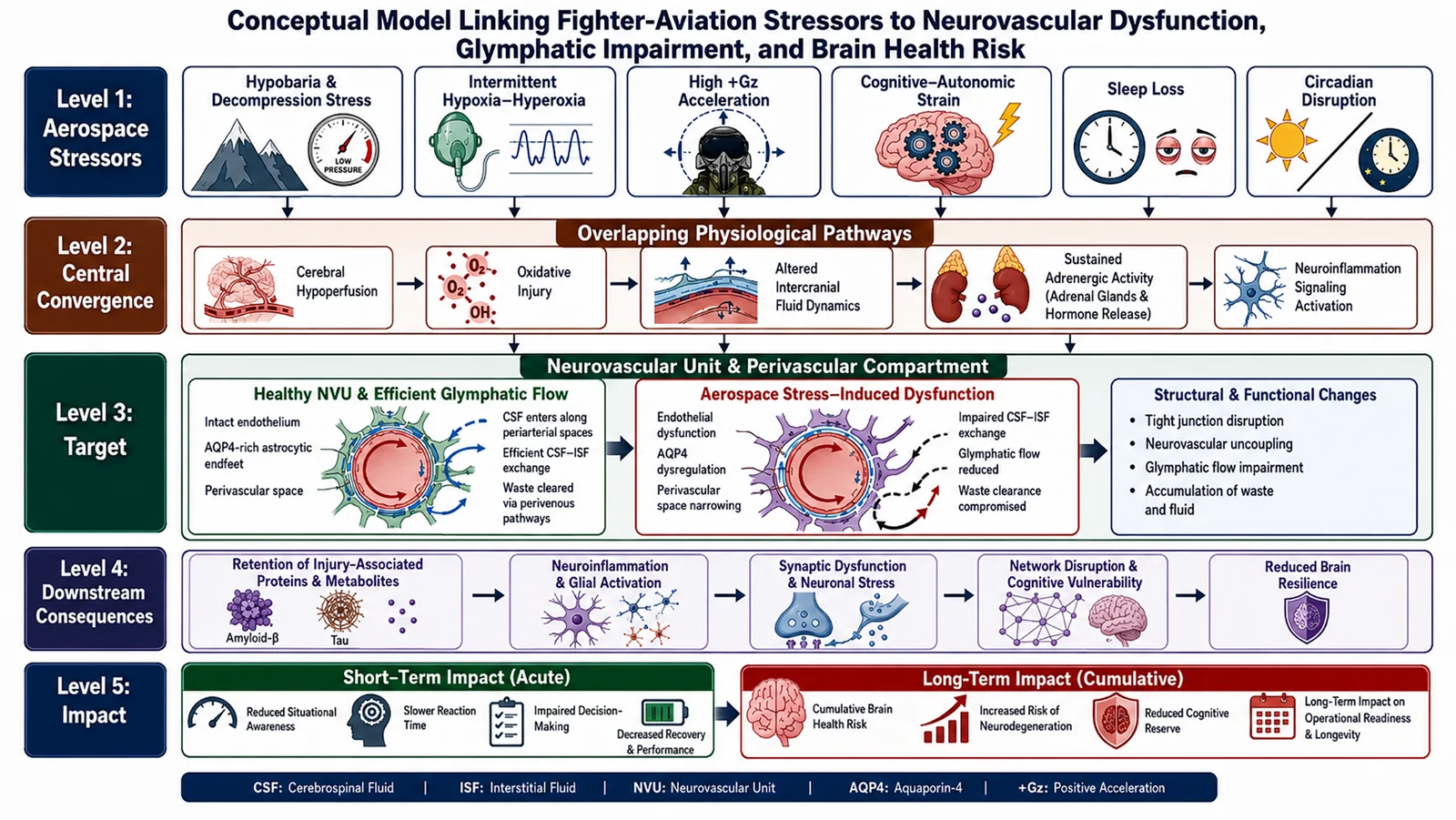
Mechanistic pathways of aerospace stressor-induced neurovascular and glymphatic dysfunction in fighter aviators. This schematic illustrates a proposed multi-level cascade by which distinct occupational exposures within the fighter jet environment may converge to culminate in acute performance decrements and cumulative long-term brain health risk. Level 1: Aerospace Stressors defines the primary operational challenges, specifically hypobaria and decompression stress, intermittent hypoxia-hyperoxia, high +Gz acceleration, cognitive-autonomic strain, sleep loss, and circadian disruption. Level 2: Central Convergence demonstrates overlapping physiological mechanisms including cerebral hypoperfusion, oxidative injury, and sustained adrenergic activation. Level 3: Target focuses on the neurovascular unit and perivascular compartment, comparing a healthy system with efficient CSF-ISF exchange and proper glymphatic flow against the dysfunctional state induced by aerospace stress. Level 4: Downstream Consequences shows the resulting retention of injury-associated proteins and metabolites and subsequent neuroinflammation. Level 5: Temporal Impact differentiates acute effects on recovery and performance from the long-term, cumulative threat to brain resilience and health. AQP4, aquaporin-4; BBB, blood-brain barrier; CSF, cerebrospinal fluid; ISF, interstitial fluid; NVU, neurovascular unit.

Controlled human altitude chamber studies provide some of the strongest evidence that repeated hypobaric decompression stress elicits measurable neurovascular and neuroinflammatory responses. Across multiple investigations, hypobaric exposure has been associated with inflammatory activation, increased circulating GFAP, and structural and metabolic brain changes despite the absence of overt neurological symptoms (Connolly et al., 2023b, 2023a, 2024). These convergent observations suggest that decompression stress produces subclinical changes that may accumulate with repeated operational exposure.

Beyond these acute physiological responses, repeated hypobaric exposure has been associated with structural brain abnormalities in some military aviation populations. Studies of U-2 pilots, altitude-chamber personnel, and RCAF aviators have reported higher WMH burden or altered white matter metrics (McGuire et al., 2013, 2014, 2016, 2019; Connolly and Lee, 2015; Ramirez et al., 2024; Ledderhos et al., 2025). A 2026 systematic review and meta-analysis of five studies (264 pilots and 177 controls) estimated higher mean WMH count and volume in pilots, but the small, heterogeneous observational evidence base cannot establish a causal effect of altitude or flight exposure (Mohamed et al., 2026). These findings support sensitive longitudinal neuroimaging and blood-based biomarker research while leaving exposure thresholds and clinical significance unresolved.

The hypobaria literature is not uniformly positive. In 20 United Kingdom altitude-chamber participants with generally brief and infrequent exposure histories, 17 had scans within the expected range and three WMH outliers had prior mild TBI; WMH burden was not related to decompression metrics, leading the authors to characterize low-intensity chamber exposure as subthreshold for promoting WMH (Connolly et al., 2018). More recent intensive chamber work has reported persistent metabolic and perfusion responses without new diffusion-weighted lesions or changes in fractional anisotropy (Connolly et al., 2024). In a separate European study of 19 altitude-chamber personnel and 28 controls, WMH count and volume rose with age and cumulative chamber time and localized cortical differences were observed, but neuropsychological and neurophysiological results did not differ; the authors found no evidence that current NATO nonhypoxic-hypobaria procedures caused brain damage (Ledderhos et al., 2025). These observations argue for dose- and context-sensitive interpretation rather than assuming that any hypobaric exposure produces cumulative structural injury.

Although the precise mechanisms remain incompletely understood, glymphatic and neurovascular dysfunction provide a plausible framework linking repeated hypobaric exposure to cumulative brain injury. Experimental and human studies suggest that decompression-related microvascular injury may compromise endothelial and pericyte integrity, disrupting components of the NVU that support both BBB function and perivascular exchange (Connolly et al., 2023b). Inflammatory responses may further alter astrocytic function and AQP4 organization, impairing the polarized water transport required for efficient glymphatic clearance, while BBB disruption may modify the pressure and permeability gradients governing CSF-ISF exchange and the transport of CNS-derived proteins into the peripheral circulation (Connolly et al., 2023b). Although these mechanisms have not been directly demonstrated in military fighter aviators, they are consistent with the broader literature linking decompression exposure to cerebral white matter abnormalities and neurovascular injury (McGuire et al., 2013; Connolly and Lee, 2015; Connolly et al., 2023b).

For clinically recognized decompression sickness (DCS), immediate oxygen and recompression with hyperbaric oxygen are established treatments (Moon, 2014). This therapeutic role should not be extrapolated to prophylaxis or chronic treatment of subclinical imaging findings. In a murine DCS model, decompression produced neuroinflammation together with increased—not reduced—glymphatic influx, illustrating that greater tracer movement can coexist with injury and does not necessarily indicate more effective recovery (Thom et al., 2023). No human evidence currently shows that hyperbaric oxygen prevents chronic glymphatic or neurovascular changes in asymptomatic aviators, and additional hyperoxia may itself impose oxidative stress (Al-Shamali et al., 2026). Future studies should distinguish emergency DCS treatment, preconditioning hypotheses, and chronic brain-health interventions.

### 3.2 Intermittent hypoxia and hyperoxia

Military fighter aviators are repeatedly exposed to fluctuations in oxygen availability resulting from high-altitude operations, supplemental oxygen systems, and rapidly changing flight conditions. These environments can produce alternating periods of intermittent hypoxia and hyperoxia that differ substantially from normal terrestrial oxygen homeostasis (Shaw et al., 2021). Recurrent oxygen fluctuations can promote oxidative stress, mitochondrial dysfunction, endothelial injury, inflammatory signaling, and impaired cerebrovascular regulation, all of which may affect BBB integrity and neurovascular function (Estrella et al., 2026). Experimental studies further demonstrate that hypobaric hypoxia exacerbates neuroinflammatory responses following TBI and worsens cerebral hypoxia during aeromedical evacuation, highlighting the potential for additive neurological stress in aviators with prior concussive or subconcussive injuries (Goodman et al., 2011; Scultetus et al., 2016).

Hyperoxia has cerebral hemodynamic effects even when arterial oxygen content is increased. In a foundational human study, Lambertsen et al. (1953) showed that oxygen breathing reduced cerebral circulation and altered cerebral metabolism, establishing oxygen-induced cerebral vasoconstriction as a longstanding physiological observation. More recently, simultaneous arterial-spin-labeling MRI, EEG, arterial blood gas, and cognitive testing demonstrated reduced global cerebral blood flow during normobaric hyperoxia alongside changes in cortical electrical activity and cognitive performance (Damato et al., 2020). These apparently divergent responses show why preserved or briefly enhanced task performance cannot be taken as evidence that cerebral perfusion is unaffected.

A randomized pCASL study subsequently characterized this response across inspired oxygen concentrations: cerebral blood flow was not significantly altered at FIO2 levels of 30–50%, but declined at concentrations of 60% or greater, supporting a dose-dependent perfusion effect (Damato et al., 2022a). In a separate normobaric and hypobaric experiment, transitions from non-steady-state oxygen exposure were reflected in arterial blood gas values, prefrontal cortical activation measured by functional near-infrared spectroscopy, and systemic cytokine profiles (Damato et al., 2024). Together, these studies indicate that inspired oxygen dose, delivery pattern, barometric pressure, and time since transition all influence the observed neurovascular response.

Oxygen-induced cerebral vasoconstriction could narrow perfusion reserve when combined with +Gz-related head-level pressure reductions or hypobaria, thereby amplifying transient neurovascular stress. That interaction is physiologically plausible but has not been established as a cause of chronic BBB or glymphatic impairment in aviators. Future studies should record FIO2 continuously, distinguish steady from rapidly changing delivery, and synchronize perfusion, EEG or fNIRS, arterial gas, inflammatory, and performance measurements across oxygen–pressure–acceleration combinations.

### 3.3 High +Gz

Military fighter aviators are routinely exposed to +Gz forces during tactical maneuvers, imposing substantial mechanical and hemodynamic stress on the cerebral circulation. Sustained or repetitive +Gz exposure redistributes blood toward the lower extremities, reducing cerebral perfusion despite the use of anti-G protective equipment and anti-G straining maneuvers. Although these countermeasures substantially reduce the risk of G-induced loss of consciousness, repeated episodes of transient cerebral hypoperfusion may still challenge cerebrovascular autoregulation, increase oxidative stress, and perturb neurovascular homeostasis (Bailey et al., 2020). Consistent with this concept, modest elevations in circulating total tau (T-tau) have been reported in RCAF aviators, potentially reflecting cumulative low-level neuronal stress associated with repeated operational exposure, while histological studies following high-altitude exposure have demonstrated tau deposition within the brain (Iacono et al., 2022; Rhind et al., 2024). Collectively, these observations support high +Gz exposure as another biologically plausible contributor to cumulative neurovascular injury over the course of an operational flying career.

EEG provides a complementary, time-resolved window on the functional consequences of acceleration-induced cerebral hypoperfusion. In centrifuge studies, Wilson et al. (2005) identified reproducible EEG changes accompanying G-induced loss of consciousness and recovery, demonstrating that transient neural consequences of perfusion failure can be detected on the time scale of the exposure. A recent conference report extended this work by examining neural markers across G-LOC and recovery (Blacker and Dooley, 2024). These studies concern overt or near-overt G-LOC and do not establish chronic injury or glymphatic dysfunction; nevertheless, they support synchronized EEG, hemodynamic, and symptom monitoring during controlled +Gz studies and, where feasible, operational research.

### 3.4 Sleep restriction and circadian disruption

Military aviators frequently experience restricted or fragmented sleep, night operations, transmeridian travel, irregular duty windows, and circadian misalignment. Section 2 summarizes the underlying sleep-dependent clearance biology; operationally, the key point is that inadequate recovery sleep can increase fatigue and cognitive vulnerability while also reducing the physiological capacity to restore neurovascular and metabolic homeostasis between sorties (Mendelsohn and Larrick, 2013; Xie et al., 2013; Hablitz et al., 2020; Hauglund et al., 2025). Sleep exposure should therefore be quantified by duration, regularity, architecture, timing, and recovery trajectory rather than treated as a binary covariate.

Aviation fatigue has a mature operational literature. The Aerospace Medical Association position paper led by Caldwell et al. (2009) synthesized the effects of sleep loss, extended wakefulness, circadian disruption, and irregular operations and emphasized layered countermeasures, with adequate sleep as the primary physiological requirement. This foundation is important for brain-health studies because fatigue is an operational endpoint with multiple causes, not a specific marker of sleep-dependent glymphatic impairment. In addition, serial study of T-6A instructor pilots linked increasing fatigue across a flying week with changing serum cytokine profiles, suggesting that systemic inflammatory fatigue may coexist with, or be partly independent of, sleepiness (Damato et al., 2022b). Future studies should therefore measure sleep physiology, workload, symptoms, and inflammatory markers rather than treating all fatigue as a single construct.

Operational use of wakefulness-promoting agents adds an important interpretive layer. Modafinil can preserve alertness during extended wakefulness (Buguet et al., 2003), and a 243-person military-aviator ground-testing series reported a low rate of acute adverse effects after a 100-mg test dose (Ooi et al., 2019). It does not, however, replace restorative sleep, and no longitudinal human study has directly tested whether chronic or repeated operational use alters glymphatic function (Rhind et al., 2001; Buguet et al., 2001). Evidence should not be presumed to indicate harm: in adolescent male rats with repetitive mild TBI, modafinil unexpectedly attenuated behavioral, metabolomic, and neuropathological abnormalities, although glymphatic transport was not directly measured and the model is not operational aviation (Christensen et al., 2024). Future cohorts should prespecify agent, dose, timing, frequency, sleep debt, and recovery sleep, and examine their relationships with DTI-ALPS, PVS, perfusion, biomarkers, and cognition.

Sleep restriction is best conceptualized as a modifiable amplifier and recovery constraint rather than a proven direct cause of aviation-related brain injury. Its effect may depend on which physiological stress preceded sleep, whether deep non-REM sleep is restored, and whether exposure recurs before vascular, inflammatory, and metabolic recovery is complete. This framing avoids attributing nonspecific fatigue or imaging findings to glymphatic failure alone and supports within-person studies that link sortie timing, sleep, physiology, and recovery over consecutive duty cycles.

### 3.5 Additional and context-dependent exposures

Noise is a persistent exposure in many flight and ground environments, with intensity and frequency spectra varying by platform, hearing protection, mission phase, and occupation. In a cross-sectional neuroimaging study of 48 men with noise-induced hearing loss and 40 controls, the noise-exposed group had lower DTI-ALPS indices and greater free-water estimates; the authors explicitly noted that the design could not establish causality (Wang et al., 2026). The study was not conducted in aviators and does not separate acoustic exposure from hearing loss, anxiety, vascular factors, or other occupational conditions. It nevertheless supports measurement of personal noise dose and auditory status in aviation cohorts rather than treating noise as background nuisance alone.

Aircrew are also exposed to cosmic ionizing radiation at altitude, with dose influenced by altitude, latitude, flight duration, solar activity, and cumulative flying time (Scheibler et al., 2022). Aviation doses and particle spectra differ substantially from deep-space exposure, so space-radiation findings cannot be transferred directly to tactical aviation. In mice, combined simulated microgravity and chronic low-dose gamma radiation altered BBB-related outcomes, whereas radiation alone did not reproduce the same pattern (Bellone et al., 2016). Direct evidence connecting aviation-relevant radiation doses to human glymphatic or meningeal-lymphatic dysfunction is absent. Radiation should therefore be treated as a measurable, context-dependent modifier and a candidate interaction term, not an established explanation for aviator WMH.

Chemical exposures are heterogeneous. PFAS may occur through firefighting foams and treated textiles; fuels, combustion products, hydraulic fluids, and solvents vary by role and airframe. A 2026 systematic review concluded that jet-fuel exposure is likely to cause human neurotoxicity under relevant conditions, particularly auditory effects, but emphasized limited high-quality epidemiology and long-term exposure data (Sprowles et al., 2026). Direct evidence for glymphatic effects is unavailable. Experimental PFOS exposure disrupts endothelial–astrocyte signaling and BBB integrity in mice (Yu et al., 2020), providing vascular plausibility rather than proof of aviator glymphatic impairment. Studies should use occupational histories, task-based sampling, validated biomonitoring, and appropriately exposed ground comparators.

### 3.6 Converging biological pathways and relative influence

The likely contribution of each stressor depends on platform, mission, intensity, timing, susceptibility, and recovery. For fighter operations, high +Gz and rapid hemodynamic transitions are the most distinctive exposures, while pressure and oxygen transitions, sleep loss, workload, and autonomic strain are plausible co-contributors. In long-duration cargo and tanker operations, circadian disruption, mission duration, and mild cabin hypobaria may be more influential than +Gz. Noise, cosmic radiation, and chemical exposures are important to measure but currently have lower and more context-dependent evidence for a glymphatic mechanism. This provisional ranking should be tested rather than treated as settled.

Interactions may be serial, additive, or synergistic. A stressor could first perturb pulmonary or systemic vascular function, a second could amplify oxidative or inflammatory signaling, and inadequate sleep could delay recovery; circulating mediators may then influence the BBB and NVU without requiring direct entry of the initiating exposure into brain tissue. Experimental hypobaric hypoxia has exacerbated neuroinflammation after TBI (Goodman et al., 2011), combined simulated microgravity and low-dose radiation produced BBB-related changes not observed with radiation alone (Bellone et al., 2016), and operational T-6A data demonstrate systemic cytokine changes across a flying week (Damato et al., 2022b). None of these studies establishes synergy among routine fighter exposures. Controlled chamber, centrifuge, oxygen, and sleep studies should isolate single effects and then test prespecified combinations, while operational longitudinal studies should estimate real-world interactions using synchronized whole-body exposure and recovery measures.

### 3.7 Platform-specific exposure phenotypes

Risk should be represented as a multidimensional exposure profile rather than a fighter-versus-nonfighter label. Fighter crews encounter the greatest repeated +Gz loads and rapid changes in perfusion, pressure, oxygen delivery, and workload. Cargo and tanker crews generally experience less +Gz but may accumulate long-duration cabin hypobaria, transmeridian circadian disruption, fatigue, and prolonged sedentary exposure. Helicopter crews usually operate at lower altitude and +Gz, often in unpressurized cabins, but can experience substantial whole-body vibration, low-frequency noise, heat, visual-motion demands, and task saturation. Pressurization and oxygen systems also vary within each category. No platform is uniformly low risk; the dominant pathway and appropriate comparator depend on the mission.

Available clinical evidence reinforces this caution. In a retrospective study of 8,598 U.S. Naval aviators, tactical aviators did not have a higher prevalence of diagnosed neurological conditions or cognitive symptoms than helicopter or reconnaissance/logistics aviators, while some behavioral-health outcomes were more frequent in non-tactical communities (Rice et al., 2026). Diagnostic records may miss subclinical imaging, biomarker, or cognitive change, but the null tactical comparison argues against assuming that high-performance flight alone determines long-term clinical risk. Future analyses should model continuous exposure components within and across platforms and should report pressurization, altitude, +Gz, vibration, noise, duty timing, and mission duration separately.

## 4 Evidence of brain injury

### 4.1 Neuroimaging markers

Neuroimaging has emerged as one of the most informative approaches for evaluating the effects of repeated aerospace exposure on the brain. Conventional structural MRI, advanced diffusion imaging, arterial spin labeling (ASL), magnetic resonance spectroscopy (MRS), and emerging glymphatic imaging techniques each provide complementary insights into brain structure, physiology, and neurovascular function (**Table 2**). To date, evidence in military aviation has been derived predominantly from structural MRI and diffusion imaging, although newer imaging approaches may further improve the detection of subclinical neurobiological changes associated with repeated operational exposure.

**Table 2:** Candidate neuroimaging and neurophysiological measures of glymphatic–neurovascular function in military aviators.

Modality/marker	Primary measures	Application in fighter cohorts	Strengths	Limitations	Reference
Structural MRI FLAIR WMH burden	Chronic white matter injury, small-vessel stress, interstitial-fluid dysregulation	Monitor longitudinal cumulative neurovascular injury	Widely available, clinically interpretable	Nonspecific mechanism	McGuire et al., 2013, 2014; Ramirez et al., 2024; Mohamed et al., 2026
PVS quantification	Enlarged perivascular spaces and impaired drainage	Assess chronic neurovascular-fluid stress	Derived from routine MRI	Nonspecific; reader variability	Wardlaw et al., 2020; Ramirez et al., 2016
DTI	White matter microstructure, axonal integrity	Detect subtle microstructural changes before gross lesions	Sensitive to subclinical injury	Not specific to glymphatic dysfunction	McGuire et al., 2016, 2019
DTI-ALPS	Indirect perivascular diffusivity and glymphatic-related transport	Estimate glymphatic-related clearance	Non-invasive, already used in human disease studies	Indirect measure; protocol-dependent	Taoka et al., 2017, 2024; Saito et al., 2023; Zahran et al., 2026
White matter free-water imaging	Extracellular free-water burden and interstitial fluid accumulation	Detect edema-like or fluid-shift related changes	Complements DTI	Can reflect multiple pathological processes	Taoka et al., 2024; Zahran et al., 2026
ASL perfusion MRI	Cerebral perfusion and blood flow	Assesses perfusion alterations related to exposure and recovery	Non-invasive perfusion assessment	Sensitive to physiology and protocol	Estrella et al., 2026
EEG	Time-resolved cortical activity, spectral and recovery dynamics	Align neural responses with +Gz, oxygen, perfusion, symptoms, and cognition	Millisecond temporal resolution; compatible with multimodal monitoring	Not a direct glymphatic or BBB measure; motion and operational artifacts	Wilson et al., 2005; Damato et al., 2020; Blacker and Dooley, 2024
Resting-state fMRI	Functional brain network connectivity	Link physiology to cognition and performance	Whole-brain functional assessment	Not specific to mechanism	Schmidt et al., 2025; Estrella et al., 2026
DCE-MRI/BBB-sensitive imaging	Barrier permeability/subtle BBB leakage	Evaluate BBB permeability	Mechanistically strong	Contrast burden, logistics, cost	Estrella et al., 2026
Phase-contrast/CSF-flow imaging	Macroscopic CSF dynamics	Characterize CSF dynamics and fluid regulation	Direct fluid-dynamics relevance	Technical complexity	Estrella et al., 2026

#### 4.1.1 White matter hyperintensities

WMH represent the most consistently reported neuroimaging abnormality in military aviation and currently provide the strongest structural evidence that repeated aerospace exposures may influence long-term brain health. Several studies involving U-2 pilots, altitude-chamber personnel, and RCAF aviators have reported increased WMH burden or altered white matter integrity despite the absence of overt neurological symptoms or clinically diagnosed decompression sickness, and a recent five-study meta-analysis found higher pooled WMH count and volume in pilots than controls (McGuire et al., 2013, 2014, 2016, 2019; Connolly and Lee, 2015; Connolly et al., 2018; Ramirez et al., 2024; Ledderhos et al., 2025; Mohamed et al., 2026). The observational designs, small source literature, and heterogeneous exposure definitions limit causal and clinical interpretation.

Although WMHs are not specific to military aviation, they are widely recognized as imaging markers of cerebral small vessel disease and have been associated with cognitive decline, impaired processing speed, executive dysfunction, and an increased risk of future neurodegenerative disease in the general population (Kloppenborg et al., 2014; d’Arbeloff et al., 2019; Duering et al., 2023). Their repeated observation across military aviation cohorts therefore raises the possibility that cumulative exposure to hypobaria, intermittent hypoxia, and other operational stressors contribute to chronic neurovascular injury, although the relative contribution of individual stressors remains uncertain.

#### 4.1.2 Perivascular spaces

When PVS become enlarged or dilated, they become visible on standard MRI. MRI-visible PVS are recognized as a neuroimaging marker of cerebral small vessel disease and candidate marker of glymphatic dysfunction. Enlargement of PVS has been associated with impaired glymphatic clearance, neurovascular dysfunction, aging, and several neurodegenerative disorders, making them an attractive imaging biomarker for investigating the long-term neurological effects of repeated aerospace exposure (Ramirez et al., 2016, 2022; Mestre et al., 2017; Rasmussen et al., 2018; Nedergaard and Goldman, 2020; Wardlaw et al., 2020).

Although direct evidence in military fighter aviators remains limited, findings from related conditions support the biological relevance of PVS as a marker of neurovascular and glymphatic dysfunction. Enlarged PVS have been reported in disorders characterized by sleep disruption, cerebral small vessel disease, and altered CSF dynamics, while spaceflight studies have similarly demonstrated PVS enlargement following prolonged exposure to microgravity (Seidler et al., 2024; Sommer et al., 2025). Furthermore, PVS burden has been associated with shorter sleep duration and greater daytime dysfunction in adults with cerebrovascular disease, further supporting a relationship between sleep physiology and MRI-visible markers of glymphatic dysfunction (Ramirez et al., 2021). Although these relationships have not yet been investigated in military fighter aviators, they provide additional biological support for the relevance of PVS as a potential imaging marker of glymphatic dysfunction.

Together, these observations suggest that PVS may provide a sensitive imaging marker for detecting cumulative neurobiological changes associated with repeated operational stress and represent a promising outcome measure for future longitudinal studies in military fighter aviators. Complementary MRI techniques capable of assessing glymphatic function more directly, such as diffusion tensor imaging along the perivascular space (DTI-ALPS), may further improve our ability to characterize these changes *in vivo*.

#### 4.1.3 Diffusion tensor imaging along the perivascular space

The DTI-ALPS index, introduced by Taoka et al. (2017), has emerged as a widely used MRI proxy for perivascular water diffusivity. Although an indirect surrogate measure, reduced DTI-ALPS indices have been associated with impaired glymphatic transport across a wide range of neurological conditions, including cerebral small vessel disease, Alzheimer’s disease, TBI, obstructive sleep apnea, and sleep disturbance (Lee et al., 2022; Roy et al., 2022; Saito et al., 2023; Mi et al., 2025; Sommer et al., 2026).

DTI-ALPS has not yet been systematically evaluated in military fighter aviators. The index is calculated in a restricted periventricular region and can be influenced by white matter geometry, lesion burden, acquisition, preprocessing, and aging; it therefore cannot be interpreted as a direct or whole-brain measure of glymphatic flow (Taoka et al., 2024; Voumvourakis et al., 2026; Zahran et al., 2026). Its operational value will depend on protocol harmonization, test–retest reliability, comparison with PVS, free-water, perfusion and sleep measures, and longitudinal association with exposure and recovery—not on a single threshold.

#### 4.1.4 Other neuroimaging and neurophysiological techniques

While most neuroimaging studies in military aviators have focused on conventional structural MRI, other modalities provide complementary information about dynamic exposure responses. ASL non-invasively quantifies cerebral blood flow and is well suited to oxygen-dose, hypobaria, +Gz, and recovery studies (Alsop et al., 2015; Damato et al., 2020, 2022a). Emerging ASL-based approaches have also been adapted to estimate clearance of labeled water following mild TBI, although this remains an indirect glymphatic measure (Joseph et al., 2024). Resting-state fMRI and MRS offer complementary assessments of network function and cerebral metabolism, while fNIRS can be deployed more flexibly during oxygen and pressure transitions (Fox and Raichle, 2007; Oz et al., 2014; Damato et al., 2024).

EEG adds millisecond temporal resolution and can detect transient cortical changes that structural MRI cannot. Its established sensitivity to G-LOC and recovery, and its simultaneous use with MRI during hyperoxia, make it particularly useful for aligning neural activity with perfusion, oxygen, symptoms, and cognitive performance (Wilson et al., 2005; Damato et al., 2020; Blacker and Dooley, 2024). EEG is not a direct measure of BBB or glymphatic integrity and should not be interpreted as a chronic injury biomarker in isolation; its value is as one component of multimodal, exposure-synchronized assessment.

### 4.2 Blood biomarkers

Blood biomarkers provide a complementary approach to neuroimaging by detecting biochemical evidence of neurological injury and physiological responses to aerospace exposure (**Table 3**). Recent advances in ultrasensitive analytical platforms, particularly single molecule array (SiMoA) technology, have enabled the quantification of brain-derived proteins that reflect complementary aspects of neuronal, astroglial, and neurovascular injury using peripheral blood samples (Khalil et al., 2018; Benussi et al., 2020; Ashton et al., 2021). These biomarkers offer the potential to identify subtle or transient neurobiological changes that may not yet be apparent on neuroimaging or clinical assessment, making them promising tools for monitoring the cumulative effects of repeated operational stress in military aviators.

**Table 3:** Candidate blood-based biomarkers of neuronal injury and neurovascular dysfunction in military aviators.

Biomarker	Primary measure	Application in fighter cohorts	Limitations
NfL, pNF-H	Neuroaxonal injury, axonal cytoskeletal disruption	Monitor cumulative axonal injury following repeated aerospace exposure	Not specific to aviation mechanism
Total tau, p-tau181, p-tau217, p-tau231	Neuronal injury, axonal stress, and tau pathology	Complement NfL for detecting subtle neuronal injury and longitudinal recovery	Interpretation requires longitudinal context
Aβ40, Aβ42, Aβ42/Aβ40	Amyloid metabolism and metabolite clearance	Investigate impaired clearance pathways and long-term neurobiological effects	Influenced by multiple factors
GFAP	Astrocytic injury	Detect astroglial responses to operational stress exposure	Best interpreted alongside other biomarkers
S100B	BBB dysfunction and astroglial injury	Assess acute neurovascular stress following aerospace exposure	May be affected by extracranial sources
PRDX-6 and related redox markers	Oxidative stress	Oxidative burden linked to hypoxia, hyperoxia, or perfusion stress	Mechanistic specificity may be limited
sPDGFRβ, ANGPT2, fibrinogen, tight-junction-related proteins, Q-albumin	Pericyte injury, endothelial permeability, barrier leakage	Useful for testing decompression-, hypoxia-, and vascular-strain hypotheses	Some require CSF or are not yet operationally mature
UCH-L1, NSE	Neuronal stress/cell-body injury	May complement axonal markers in acute studies	May show limited sensitivity in chronic low-level exposure
Cytokines, chemokines, extracellular vesicles/microparticles	Systemic inflammation and vascular activation; potential peripheral-to-CNS signaling	Track whole-body inflammatory responses across flying schedules and relate them to fatigue, perfusion, sleep, BBB-sensitive imaging, and CNS biomarkers (Damato et al., 2022b)	Nonspecific and temporally labile; elevation does not prove BBB passage, neuroinflammation, or glymphatic dysfunction

Neurofilament light chain (NfL) is one of the best-established blood biomarkers of neuroaxonal injury and a sensitive indicator of axonal damage across a wide range of neurological disorders, including TBI, multiple sclerosis, Alzheimer’s disease, and other neurodegenerative diseases (Khalil et al., 2018; Bridel et al., 2019). In military aviation, elevations in circulating NfL following repeated aerospace exposure suggest that operational stressors may produce subtle neuroaxonal injury even in the absence of clinically apparent neurological deficits (Rhind et al., 2024). Tau, a microtubule-associated protein involved in axonal stability, has also been investigated as a marker of neuronal injury. While circulating tau concentrations are generally lower and more variable than NfL, reported elevations following acute neurological injury suggest that tau may provide complementary information regarding neuronal damage associated with repeated aerospace exposure (Zetterberg and Blennow, 2016; Ashton et al., 2021; Iacono et al., 2022).

Beyond neuroaxonal injury, several circulating biomarkers have been investigated as indicators of astrocytic, neurovascular, and oxidative stress responses to repeated aerospace exposure. Among these, GFAP has emerged as a sensitive marker of astrocytic injury, whereas S100 calcium-binding protein B (S100B) has been widely used as a surrogate marker of BBB permeability and astroglial injury (Yang and Wang, 2015; Abdelhak et al., 2022). Biomarkers of oxidative stress, including peroxiredoxin-6 (PRDX-6), may provide additional insights into the biological consequences of repeated aerospace exposure, with recent evidence demonstrating elevated circulating PRDX-6 levels in military aviators (Rhind et al., 2024). Although these biomarkers have demonstrated utility across a range of neurological disorders, evidence in military aviators remains limited. Preliminary findings suggest transient astrocytic, neurovascular, and oxidative stress responses following repeated aerospace exposure; however, larger independent studies are needed to establish their sensitivity, specificity, and long-term clinical significance.

Operational evidence supports adding systemic immune markers to this panel. Damato et al. (2022b) studied 22 T-6A instructor pilots who completed at least two sorties and obtained serial samples across a week-long flying schedule. Twenty of 37 measurable serum analytes changed from baseline, 13 pilots reported increased fatigue, and 11 analytes were associated with increasing general-fatigue scores. The small cohort, absence of a non-flying control group, and multiple concurrent exposures prevent attribution to a specific stressor or inference of BBB injury. The study nevertheless demonstrates that high-performance aviation can be accompanied by measurable whole-body inflammatory and vascular signaling, providing a direct rationale for pairing cytokines with sleep, perfusion, BBB-sensitive imaging, and CNS-derived biomarkers.

Supporting evidence from saturation divers, whose repeated decompression exposures share physiological similarities with aviation-related pressure stress, has likewise demonstrated post-exposure elevations in NfL and S100B, consistent with neuroaxonal and astroglial injury (Rosén et al., 2020).

Given the complexity of neurological injury, a multi-marker approach may provide a more comprehensive assessment than any single biomarker alone by capturing complementary aspects of neuronal, astroglial, neurovascular, and oxidative stress responses. Similar biomarker panels have demonstrated prognostic utility in TBI, supporting the potential value of integrated biomarker strategies for monitoring the cumulative neurological effects of repeated aerospace exposure in military aviators (Di Battista et al., 2015).

Collectively, the available evidence suggests that repeated aerospace exposure elicits measurable neurobiological responses that can be detected using circulating blood biomarkers. Although many reported biomarker changes are transient and their long-term clinical significance remains uncertain, advances in ultrasensitive analytical platforms provide a promising, minimally invasive approach for monitoring the cumulative neurological effects of repeated aerospace exposure and evaluating potential countermeasures in military aviators.

An important consideration for future biomarker studies is that brain-to-blood transport may influence the appearance of CNS-derived proteins in peripheral blood. In a randomized crossover study of 39 healthy older adults, Dagum et al. (2026) found higher morning plasma Aβ and tau species after normal sleep than after sleep deprivation; investigational physiological measurements and multicompartment modeling were consistent with enhanced sleep-active clearance. Because glymphatic transport was inferred rather than directly quantified with a validated tracer, the study is mechanistically supportive rather than definitive. Sleep restriction and circadian disruption may therefore alter circulating biomarker concentrations independently of the underlying degree of neurological injury, underscoring the importance of standardized sampling time and sleep phenotyping in military aviators.

### 4.3 Cognitive outcomes

Ultimately, the clinical relevance of neuroimaging and blood biomarker abnormalities depends on whether repeated aerospace exposure is associated with measurable changes in cognitive function. Although military aviators generally maintain the high levels of cognitive performance required for operational readiness, several studies have reported subtle differences in attention, processing speed, working memory, and executive function following repeated exposure to hypobaric, hypoxic, and acceleration-related stressors (McGuire et al., 2013, 2014). However, findings remain inconsistent, likely reflecting differences in study design, exposure history, participant characteristics, and cognitive assessment methods.

Attention and processing speed are among the most frequently evaluated cognitive domains because they are critical for flight performance and operational decision-making. Although military aviators generally continue to perform within normal cognitive ranges, several studies have reported subtle reductions in processing speed, sustained attention, and psychomotor performance following repeated aerospace exposure (McGuire et al., 2013, 2014). However, these effects are generally small and often transient, making it difficult to distinguish the consequences of operational exposure from those of fatigue, sleep disruption, training, and individual variability.

Evidence from neuroimaging and blood biomarker studies further suggests that structural and molecular markers of brain injury may be associated with cognitive function. Across diverse clinical and research populations, neuroimaging measures of cerebral small vessel disease and glymphatic dysfunction, together with circulating biomarkers of neuroaxonal and astroglial injury, have been associated with cognitive performance (Andriuta et al., 2024). However, these relationships remain incompletely understood, and few studies have evaluated these complementary measures simultaneously in military aviators.

Importantly, available evidence suggests that these neurobiological changes may remain largely subclinical. In a retrospective cohort study of 8,598 US Naval aviators spanning tactical (F/A-18 and F-35), helicopter, and reconnaissance/logistics communities, tactical aviators did not demonstrate a higher prevalence of diagnosed neurological disorders or cognitive symptoms than their non-tactical counterparts despite repeated operational exposure (Rice et al., 2026). However, the authors acknowledged that healthcare avoidance, reported by more than 70% of military pilots, together with the inherent limitations of retrospective electronic health record analyses, likely underestimated the true prevalence of neurological disease. They therefore called for prospective longitudinal studies incorporating biomarkers and neuroimaging (Rice et al., 2026). Collectively, these findings suggest that subtle neurobiological changes may accumulate long before overt clinical impairment becomes apparent, emphasizing the need for prospective longitudinal studies integrating cognitive assessment, neuroimaging, and blood biomarkers.

Although current evidence does not demonstrate a higher prevalence of clinically apparent neurological disease among military aviators, the long-term consequences of repeated aerospace exposure remain uncertain. Experimental and neuropathological evidence demonstrating tau deposition, neuroinflammation, and myelin loss following high-altitude exposure, together with broader evidence linking occupational vascular exposures to cerebral small vessel disease and vascular dementia, underscores the importance of continued surveillance and prospective longitudinal investigation of cognitive health in military aviation populations (Iacono et al., 2022; Clancy et al., 2024).

## 5 Lessons from adjacent fields

### 5.1 Spaceflight as an analog model

Although microgravity differs fundamentally from military flight, spaceflight provides a valuable human model demonstrating that sustained perturbations of intracranial fluid dynamics can produce measurable neurovascular, glymphatic, and biomarker changes. Long-duration spaceflight has been associated with increased circulating NfL, total tau, and GFAP, consistent with neuroaxonal injury and astroglial activation, as well as increased WMH burden on MRI (Schmidt et al., 2025; Zu Eulenburg et al., 2021; Alperin et al., 2017). A recent Lancet Neurology review synthesized growing evidence that long-duration spaceflight alters intracranial fluid dynamics, including ventricular expansion (7–25%), upward brain displacement, basal ganglia PVS enlargement, and stagnant or retrograde jugular venous flow (Seidler et al., 2024). Together, these findings support widespread alterations in CSF circulation and glymphatic function. Notably, post-flight increases in circulating Aβ were interpreted as a potential washout phase following impaired glymphatic clearance during microgravity. Importantly, cognitive effects may not manifest until crew members reach older age, when glymphatic clearance naturally slows, highlighting a potential long-latency consequence directly relevant to career-long monitoring of military aviators. Collectively, these observations suggest that disturbances in intracranial fluid homeostasis can produce detectable structural, physiological, and molecular signatures even in otherwise healthy individuals.

In the 30-day NASA–DLR SANS Countermeasure (SANS CM) study, Schmidt et al. (2025) measured plasma NfL, GFAP, Aβ40, Aβ42, and total tau before, during, and after 30 days of head-down tilt bed rest (HDTBR). Despite the absence of overt neurological impairment, participants exhibited persistent changes in multiple CNS biomarkers, suggesting that physiological alterations in intracranial fluid dynamics can produce measurable biomarker responses while remaining clinically silent. These findings closely parallel observations in RCAF aviators and support the concept that circulating biomarkers may reflect both neurobiological stress and altered glymphatic clearance dynamics (Rhind et al., 2024; Schmidt et al., 2025).

Beyond biomarker changes, spaceflight analog studies demonstrate widespread alterations in neurovascular physiology, including cerebral hemodynamics, intracranial fluid regulation, and BBB integrity, with several adaptations showing reversibility following return to Earth (Estrella et al., 2026). This reversibility suggests that at least some neurovascular changes are dynamic rather than permanent, making spaceflight research particularly informative for understanding the mechanisms and time course of recovery following repeated aerospace exposures. Although fighter aviation and spaceflight expose individuals to fundamentally different environmental conditions, both repeatedly challenge neurovascular and intracranial fluid homeostasis. Consequently, spaceflight provides converging evidence that repeated disruption of these systems can produce the structural, physiological, and molecular changes hypothesized to contribute to long-term brain health in military aviators.

### 5.2 Traumatic brain injury as an analog model

Although TBI differs fundamentally from routine military flight, it provides one of the strongest human models linking neurovascular injury, glymphatic dysfunction, and impaired brain waste clearance. Experimental and clinical studies demonstrate that glymphatic transport is impaired following TBI, in part because AQP4 channels become mislocalized away from astrocytic endfeet, reducing the efficiency of perivascular fluid exchange (Sullan et al., 2018). Iliff et al. (2014) further demonstrated that AQP4 mislocalization directly impairs glymphatic function and promotes tau pathology after TBI, establishing a mechanistic link between post-injury clearance failure and neurodegenerative protein accumulation. Consistent with these findings, a systematic review concluded that glymphatic dysfunction is a common feature across the spectrum of TBI severity, with AQP4 disruption and sleep disturbance identified as major contributors to impaired waste clearance (Ferrara et al., 2022).

Sleep disturbance represents an important mediator of glymphatic dysfunction following TBI. TBI disrupts normal sleep architecture, while impaired sleep further reduces glymphatic clearance, creating a bidirectional cycle that may promote protein aggregation and persistent neurological dysfunction (Piantino et al., 2019, 2022). Consistent with this model, mild TBI, poor sleep quality, and MRI-visible PVS co-occur among military veterans in a pattern suggestive of impaired glymphatic function (Piantino et al., 2021). Longitudinal neuroimaging studies further demonstrate progressive PVS enlargement and declining DTI-ALPS indices following mild TBI, with baseline glymphatic measures predicting subsequent cognitive recovery (Zhuo et al., 2024). Together, these findings suggest that impaired glymphatic clearance may contribute to the accumulation of Aβ and tau after injury, providing one potential mechanism linking acute TBI with later neurodegeneration (Peters and Lyketsos, 2023). Supporting this hypothesis, experimental studies demonstrate that meningeal lymphatic dysfunction exacerbates TBI pathology, whereas restoration of lymphatic drainage improves neurological outcomes (Bolte et al., 2020; Barker et al., 2025).

Although routine military aviation is not equivalent to overt TBI, repeated exposure to acceleration, vibration, hypobaria, and, in some operational settings, blast may engage overlapping neurovascular and glymphatic pathways. Reviews of military-related mild TBI have identified advanced neuroimaging approaches, including DTI-ALPS and perivascular space quantification, as promising tools for detecting microstructural abnormalities not evident on conventional MRI (Kim et al., 2023). Experimental blast models demonstrate disruption of AQP4 organization together with widespread neuropathological changes affecting glymphatic and neurovascular function (Iacono et al., 2023; Braun et al., 2024). Human studies of experienced military breachers have likewise reported altered circulating myeloperoxidase, matrix metalloproteinases, and neurovascular endothelial molecules following repetitive low-level blast exposure (Rhind et al., 2025). Consistent with these findings, human neuroimaging studies report abnormalities in DTI-ALPS, enlarged PVS, ASL perfusion measures, and other markers of glymphatic dysfunction across the spectrum of TBI severity and chronicity (Christensen et al., 2020; Dai et al., 2023; Park et al., 2023; Joseph et al., 2024; Yang et al., 2024, 2026; Michalaki et al., 2025; Miettinen et al., 2025; Broomand Lomer et al., 2026).

Comparable glymphatic and cognitive alterations have also been reported following repetitive sub-concussive head impacts in contact-sport athletes (Morita et al., 2023; Gay et al., 2025). Similarly, cumulative blast exposure models developed for Special Operations Forces provide methodological frameworks that could be adapted to quantify lifetime aerospace exposure in military aviators, while greater perivascular space burden has been associated with both career stage and mild TBI history in Special Operations personnel (McEvoy et al., 2023; Powell et al., 2024). Collectively, these findings suggest that glymphatic dysfunction may represent a common downstream response to repeated neurophysiological stress, supporting investigation of similar mechanisms in military aviation.

### 5.3 Systemic inflammation and cerebral small vessel disease

Cerebral small vessel disease (CSVD) offers an adjacent clinical model in which systemic inflammation, neurovascular coupling, BBB integrity, PVS, WMH, and candidate glymphatic markers can be examined together. Recent reviews describe a reciprocal cycle: peripheral or central inflammation may disrupt astrocytic AQP4 organization and perivascular transport, while impaired clearance may retain inflammatory mediators and pathological proteins (Cai et al., 2024; Zou et al., 2024). A broader neurovascular–glymphatic synthesis similarly places BBB breakdown, pericyte dysfunction, altered perfusion, and impaired meningeal or perivascular clearance within an interacting cascade rather than competing single mechanisms (Mansour et al., 2025).

In a prospective multimodal MRI study of 155 patients with CSVD and 70 healthy controls, Chen et al. (2026) reported lower neurovascular coupling, lower DTI-ALPS indices, and greater choroid-plexus and PVS fractions in the CSVD group. Structural-equation modeling associated inflammatory biomarkers with vascular lesion burden both directly and indirectly through glymphatic and serial neurovascular-coupling–glymphatic pathways; greater vascular burden was associated with poorer cognition. Because the study was observational and conducted in a clinical CSVD population, the modeled pathways do not prove mediation in aviators. They do, however, provide a concrete human template for testing systemic inflammatory, neurovascular, clearance, WMH, and cognitive measures in the same longitudinal aerospace cohort.

### 5.4 High-altitude physiology and cerebral fluid homeostasis

Complementing findings from spaceflight and TBI, high-altitude research provides a useful translational bridge by examining the combined effects of hypobaria, hypoxia, cerebral edema risk, and sleep disturbance on intracranial fluid homeostasis. A glymphatic hypothesis has been proposed in the context of high-altitude cerebral edema (HACE), emphasizing the roles of AQP4, cerebral venous drainage, and sleep-related physiology (Simka et al., 2018). Their hypothesis proposes that water enters the brain primarily through AQP4 water channels, that acetazolamide may exert part of its protective effect through inhibition of AQP4, and that sleep at high altitude is a recognized risk factor for HACE, collectively supporting a role for glymphatic dysfunction (Kamegawa et al., 2016; Simka et al., 2018). Accordingly, they identified several interventions known to influence glymphatic function as priorities for future investigation, including modulation of the noradrenergic system, sleep posture, cerebral venous anatomy, and physical activity (Simka et al., 2018). Although military aviation differs from sustained high-altitude exposure, both environments involve hypobaric stress, oxygen perturbation, and altered intracranial fluid dynamics that may contribute to similar neurovascular and glymphatic responses.

## 6 Translational research agenda for next-generation military aviation cohorts

### 6.1 Core hypothesis

The central hypothesis emerging from this review is that repeated military aviation exposure may alter neurovascular and perivascular homeostasis through combinations of cerebral perfusion change, BBB and endothelial dysfunction, astrocytic responses, oxidative and inflammatory stress, autonomic strain, and impaired sleep-dependent recovery. Glymphatic dysfunction is treated here as a candidate contributing mechanism within that network, not as the sole or presumed primary pathway. Multimodal neuroimaging, fluid biomarkers, physiological monitoring, and exposure-resolved analyses may detect changes before overt neurological disease, but longitudinal evidence is required to determine whether those changes are persistent, reversible, clinically meaningful, or causally related to flight. **Figure 2** presents a candidate multimodal longitudinal monitoring framework for testing these relationships.

**Figure 2:**
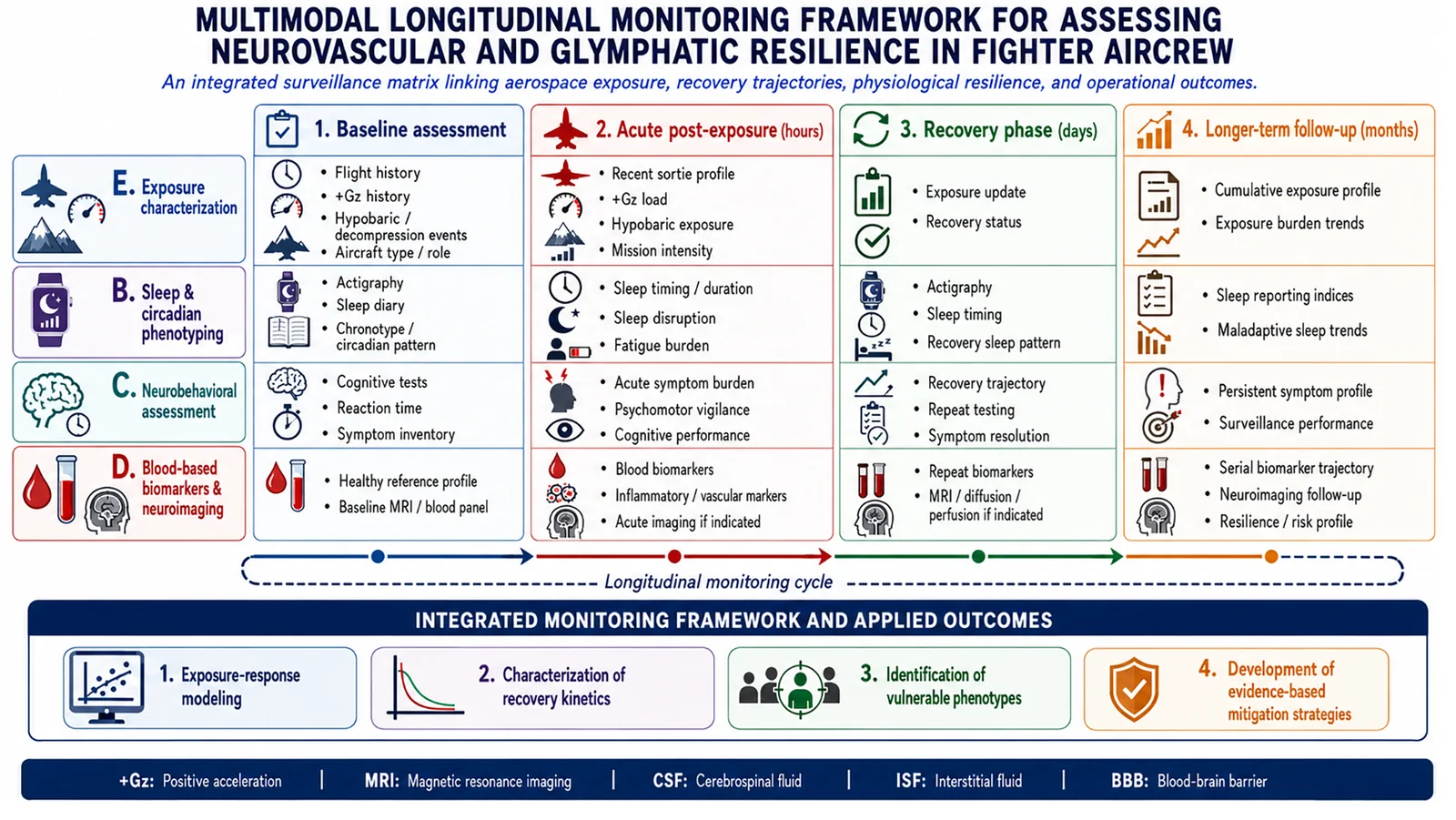
Candidate multimodal longitudinal monitoring framework for assessing neurovascular and glymphatic resilience in fighter aircrew. This image outlines a comprehensive strategy for next-generation pilot health surveillance, structured as an integrated matrix designed to quantify the relationship between stress, recovery, and individual vulnerability. The monitoring timeline is organized across four critical, repeating phases: baseline assessment, acute post-exposure (immediately following flight operations), recovery phase (days), and longer-term follow-up (months). Across these longitudinal timepoints, the framework simultaneously captures four distinct but complementary data streams: (1) detailed exposure characterization tracking flight hours, +Gz loads, and hypobaric events; (2) objective sleep and circadian phenotyping utilizing actigraphy and diaries; (3) precise neurobehavioural assessment including objective cognitive and reaction time testing; and (4) blood-based biomarkers and advanced neuroimaging providing cellular and structural insight. DTI-FW, Diffusion Tensor Imaging-Free Water; GFAP, Glial Fibrillary Acidic Protein; NfL, Neurofilament Light chain; pCASL, pseudo-Continuous Arterial Spin Labeling.

### 6.2 Key study designs

Testing the central hypothesis will require complementary designs across timescales and operational contexts. Cross-sectional studies can define broad phenotypes but should stratify by platform and model continuous exposure components rather than using fighter status as the sole proxy. Prospective cohorts should begin before advanced-airframe conversion, repeat measurements after defined exposure blocks and recovery intervals, and include matched cargo/tanker, helicopter, ground-crew, and remotely piloted aircraft (RPA) groups. RPA operators omit direct +Gz, hypobaria, vibration, and cockpit oxygen transitions while retaining shift work, sustained vigilance, workload, and operational stress; they are therefore a useful mechanistic comparison, not an unexposed control. In 366 air personnel undergoing sleep-deprivation testing, both RPA operators and manned aircrew deteriorated after 20 hours awake, although group trajectories differed (Algranati et al., 2024). Acute pre/post-sortie studies should quantify reversibility, while career-long studies should estimate accumulation. Across designs, synchronized flight-data records, oxygen and pressure histories, EEG or deployable neurophysiology where feasible, cardiorespiratory and whole-body physiological measures, personal noise and radiation dosimetry, sleep and stimulant logs, vascular risk factors, and environmental-exposure measures should be linked to imaging, blood biomarkers, cognition, and symptoms.

### 6.3 Gaps and future directions

#### 6.3.1 Stress biology and glymphatic resilience

Recent advances in glymphatic research increasingly support the concept that the brain’s waste clearance system functions as a mediator of physiological resilience rather than solely a mechanism of waste removal. Emerging evidence suggests that both acute and chronic stress disrupt astrocytic function, particularly AQP4 channel polarization, impair metabolic waste clearance, and contribute to cognitive decline and psychiatric vulnerability. A recent study demonstrated that gBOLD-CSF coupling transiently decreases during acute psychosocial stress in healthy adults before returning to baseline following a 60-minute recovery period (Jin et al., 2025). Importantly, the magnitude of this stress-induced glymphatic fluctuation was positively associated with both trait psychological resilience and the cortisol awakening response, positioning the glymphatic system as a dynamic component of an integrated neuroendocrine stress response. These findings extend the glymphatic framework beyond chronic injury, suggesting that individuals with greater glymphatic plasticity may recover more efficiently from acute physiological stress.

#### 6.3.2 Potential countermeasures

Near-term countermeasures should target exposures and recovery processes with established operational relevance: adequate oxygenation and pressure protection, optimized hearing protection, sleep and circadian scheduling, fatigue-risk management, fitness, vascular-risk reduction, and structured post-sortie recovery. Wakefulness-promoting agents may preserve performance in selected circumstances but should be studied alongside—not substituted for—recovery sleep. Candidate glymphatic-directed interventions such as exercise, respiratory protocols, acoustic or photobiomodulation approaches, and neuromodulation remain experimental for this population and require safety, dose, and mechanism testing before operational adoption (He et al., 2017; Salehpour et al., 2022; Van den Bulcke and Van Den Bossche, 2024; Sundman et al., 2025).

#### 6.3.3 Priority research gaps

The first priority is causal decomposition. Factorial and crossover experiments should isolate hypobaria, oxygen transitions, +Gz, sleep restriction, noise, and their prespecified combinations under ethically controlled conditions, with sufficient recovery sampling to detect delayed or reversible responses. These studies should test whether combined effects are additive, subadditive, or synergistic and should explicitly include null hypotheses. The second priority is exposure-resolved operational research across fighter, cargo/tanker, helicopter, and RPA communities, with within-platform variability in pressurization, mission duration, workload, and protective systems. The third is validation of glymphatic surrogates: DTI-ALPS, PVS, free-water, perfusion, and circulating biomarkers must be compared with one another and interpreted against vascular, sleep, auditory, and environmental covariates. Finally, sex, age, vascular risk, prior TBI, susceptibility to DCS, stimulant use, recovery sleep, PFAS and other chemical biomarkers, personal noise dose, and radiation dose should be incorporated prospectively rather than addressed only after associations emerge.

#### 6.3.4 Therapeutic opportunities

Intervention studies should first establish whether an exposure-linked abnormality is present, reproducible, and reversible. Hyperbaric oxygen should remain clearly separated into three questions: it is established treatment for clinical DCS; its prophylactic use before or after routine flight is unproven; and its value for chronic, subclinical neuroimaging or biomarker changes is unknown (Moon, 2014). The murine finding of increased glymphatic influx during DCS-associated neuroinflammation further cautions against interpreting increased transport as uniformly beneficial (Thom et al., 2023). Trials of sleep, circadian, exercise, respiratory, or other interventions should therefore include clinical outcomes, adverse effects, mechanistic biomarkers, and durability rather than using a single putative clearance measure as the endpoint.

### 6.4 Limitations and caution in interpretation

A major limitation is the absence of direct evidence linking military flight exposure to glymphatic dysfunction. The framework is inferential and draws on heterogeneous human, animal, imaging, and adjacent-field evidence. Glymphatic physiology itself remains actively debated, particularly the routes and driving forces of parenchymal solute transport, and common human measures such as PVS and DTI-ALPS are indirect and nonspecific (Abbott et al., 2018; Smith and Verkman, 2018; Voumvourakis et al., 2026; Wang et al., 2026). Likewise, systemic cytokines, EEG changes, and perfusion reductions are not specific markers of BBB disruption or glymphatic failure. Consequently, glymphatic change cannot presently be designated the sole or primary mechanism; whole-body cardiopulmonary and immune responses, direct tissue injury, perfusion change, BBB dysfunction, oxidative stress, autonomic effects, and sleep-related recovery may provide independent or interacting explanations.

The evidence base is also vulnerable to selection, confounding, small samples, exposure misclassification, and publication bias. RCAF biomarker findings are cross-sectional and cannot distinguish acute responses, cumulative exposure, pre-existing differences, or susceptibility (Ramirez et al., 2024; Rhind et al., 2024). Low-intensity altitude-chamber exposure was not associated with WMH in most participants (Connolly et al., 2018), and tactical aviators did not show greater diagnosed neurological morbidity than non-tactical peers in a large retrospective cohort (Rice et al., 2026). Noise, radiation, PFAS, and modafinil evidence is indirect or context-specific. Most studies include few women and provide limited information on platform, pressurization, dose, recovery, hearing, vascular risk, or co-exposures. Narrative selection without formal risk-of-bias grading further limits certainty.

Accordingly, this review does not conclude that military aviation has been shown to impair glymphatic function or that any observed WMH, biomarker change, or cognitive outcome is caused by impaired clearance. It proposes a calibrated, testable glymphatic–neurovascular framework in which causal alternatives, null findings, platform-specific exposure mixtures, and recovery processes are evaluated prospectively.

## 7 Conclusions

Military aviation exposes aircrew to platform- and mission-specific combinations of physiological, operational, and environmental stressors. Glymphatic dysfunction is one plausible contributor within a broader neurovascular and recovery network, but direct evidence in aviators is not yet available and current findings include important null and equivocal results. Fighter, cargo/tanker, helicopter, and RPA communities should therefore not be collapsed into a single exposure hierarchy; each offers a distinct combination of +Gz, pressure, oxygen, vibration, noise, sleep, workload, radiation, and chemical context.

Prospective, longitudinal research that links synchronized exposure measurements to sleep and circadian phenotyping, multimodal neuroimaging, fluid biomarkers, cognition, and recovery is now feasible. Controlled single- and combined-stressor experiments can establish relative influence and interaction, while platform-resolved cohorts and RPA comparisons can test operational generalizability. This evidence is needed before glymphatic biomarkers or targeted interventions can inform clinical surveillance, countermeasure policy, or long-term risk communication for military aviators.
